# Predictors of long-term cognitive outcome in Alzheimer's disease

**DOI:** 10.1186/alzrt85

**Published:** 2011-07-20

**Authors:** Carina Wattmo, Åsa K Wallin, Elisabet Londos, Lennart Minthon

**Affiliations:** 1 Clinical Memory Research Unit, Department of Clinical Sciences, Malmö, Lund University, SE-205 02 Malmö, Sweden; 2 Department of Neuropsychiatry, Skåne University Hospital, SE-205 02 Malmö, Sweden

## Abstract

**Introduction:**

The objective of this study was to describe the longitudinal cognitive outcome in Alzheimer's disease (AD) and analyze factors that affect the outcome, including the impact of different cholinesterase inhibitors (ChEI).

**Methods:**

In an open, three-year, nonrandomized, prospective, multicenter study, 843 patients were treated with donepezil, rivastigmine, or galantamine in a routine clinical setting. At baseline and every six months, patients were assessed using several rating scales, including the Mini-Mental State Examination (MMSE) and the Alzheimer's Disease Assessment Scale-cognitive subscale (ADAS-cog) and the dose of ChEI was recorded. Sociodemographic and clinical characteristics were investigated. The relationships of these predictors with longitudinal cognitive ability were analyzed using mixed-effects models.

**Results:**

Slower long-term cognitive decline was associated with a higher cognitive ability at baseline or a lower level of education. The improvement in cognitive response after six months of ChEI therapy and a more positive longitudinal outcome were related to a higher mean dose of ChEI, nonsteroidal anti-inflammatory drug (NSAID)/acetylsalicylic acid usage, male gender, older age, and absence of the apolipoprotein E (APOE) ε4 allele. More severe cognitive impairment at baseline also predicted an improved response to ChEI treatment after six months. The type of ChEI agent did not influence the short-term response or the long-term outcome.

**Conclusions:**

In this three-year AD study performed in a routine clinical practice, the response to ChEI treatment and longitudinal cognitive outcome were better in males, older individuals, non-carriers of the APOE ε4 allele, patients treated with NSAIDs/acetylsalicylic acid, and those receiving a higher dose of ChEI, regardless of the drug agent.

## Introduction

Alzheimer's disease (AD) is the most prevalent cause of dementia among the elderly, accounting for 50% to 60% of cases [1]. This progressive neurodegenerative disease affects approximately 24 million individuals worldwide, with one new case detected every seven seconds [2]. AD patients exhibit the following symptoms: decline in executive functions, memory impairment, visuospatial and language difficulties, and behavioral disturbances [3].

The loss of cholinergic transmission is assumed as one of the causes of the cognitive deterioration detected in patients with AD [4]. Based on this cholinergic hypothesis, several acetylcholinesterase inhibitors (ChEIs) have been introduced as treatments for AD. The ChEIs available currently (that is, donepezil, rivastigmine, and galantamine) yielded modest improvements in cognition and global performance compared with placebo treatment in subjects with varying degrees of AD severity. The benefits of this treatment regarding activities of daily living (ADL) and behavior were also observed [5,6].

However, not every patient benefits from ChEI treatment. The heterogeneity in cognitive outcome and response to treatment emphasize the importance of identifying patients who respond positively to the treatment, to enhance the drug's efficacy and its cost benefits in AD [7].

No prospective head-to-head studies of ChEI therapy in AD longer than two years have been published. Two long-term randomized studies have been reported: a two-year trial of donepezil vs rivastigmine [8] and a one-year comparison of donepezil and galantamine [9]. The three drug agents were compared in several naturalistic six- to nine-month studies from the Italian Chronos project [10-12] and in one study from Spain [13]. Regarding cognition, all but one study found no differences between the drugs. A 12-week open-label trial showed that donepezil was superior to galantamine [14]. Conflicting results concerning ADL have been described [8,10,14].

The longitudinal course of AD is complex and several sociodemographic and clinical factors, such as younger age or higher education [15,16], being a carrier of the apolipoprotein E (APOE) ε4 allele [17], or moderate-to-severe level of dementia [15,18] have been suggested to increase the rate of cognitive decline in untreated patients. Other studies showed that these variables had no effect on disease progression: age [16], education [19], presence of the APOE ε4 allele [20], or level of dementia [21]. An improved response to ChEI treatment was observed in patients who were more cognitively impaired [7,22]. Inconsistent results were found regarding gender [23,24] and age [10,25]. The divergent results of these studies imply that the influence of these factors needs further investigation. Advanced multivariate methods can provide a clearer pattern of the complex impact of predictors.

In this study, we used mixed-effects models (linear and nonlinear) to achieve a higher resolution in the analysis of the long-term association between potential predictive characteristics, including a comparison of the three ChEI agents, on the cognitive outcome of AD patients in a routine clinical setting.

The aims of this study were: 1) to identify the sociodemographic and clinical factors that influence the longitudinal cognitive outcome and response to ChEI treatment, and 2) to study the impact of different ChEI agents and dosages.

## Materials and methods

### Study and subjects

The Swedish Alzheimer Treatment Study (SATS) was started to investigate the long-term efficacy of ChEI treatment in naturalistic AD patients in clinical practice. SATS is a three-year, open-label, observational, nonrandomized, multicenter study that was described in detail previously [26]. Its purpose is the evaluation of cognition, global performance, and ADL every six months. The subjects were prospectively recruited from 14 memory clinics located in different areas of Sweden. Most participants are in the mild-to-moderate stages of the disease and the SATS is still ongoing. All subjects exhibiting a baseline Mini-Mental State Examination (MMSE) [27] score ranging from 10 to 26 and for whom at least three measurements were available per individual (to model nonlinearity in the trajectories better) [28,29] were included in this study. A total of 843 patients (donepezil, *n *= 456; rivastigmine, *n *= 183; and galantamine, *n *= 204) who were enrolled until the end of December 2005 fulfilled these criteria, thus having the opportunity to complete the full three-year SATS program.

Outpatients aged 40 years and older who met the criteria for the clinical diagnosis of dementia, as defined by the *Diagnostic and Statistical Manual of Mental Disorders*, 4^th ^edition (DSM-IV) [30], and for possible or probable AD, according to the criteria of the National Institute of Neurological and Communicative Disorders and Stroke and the Alzheimer's Disease and related Disorders Association (NINCDS-ADRDA) [31], were considered for inclusion. All patients were diagnosed by physicians specialized in dementia disorders. Moreover, the selected patients had to live at home at the time of diagnosis, have a responsible caregiver, and be assessable with the MMSE at the start of the ChEI treatment (baseline). After the baseline assessments, patients were prescribed a ChEI treatment according to the approved product labeling and paid for their own medication, as in a routine clinical practice. The choice of drug and dosage for the individual patient was left entirely up to the physician's discretion and professional judgment. Medications other than anti-dementia drugs were allowed and documented during the study. Reasons for study withdrawal were recorded and presented for this cohort of patients. Nursing-home placement was not a reason for dropout if the patient was able to continue to visit the clinic.

All patients and/or caregivers provided informed consent to participate in the study, which was conducted according to the provisions of the Helsinki Declaration and was approved by the Ethics Committee of Lund University, Sweden.

### Outcome measures

Cognitive ability was assessed using the MMSE, with scores ranging from 0 to 30 (a lower score indicating more impaired cognition), and the Alzheimer's Disease Assessment Scale-cognitive subscale (ADAS-cog) [32], with a total range of 0 to 70 (a higher score indicating a more impaired cognition).

The Instrumental Activity of Daily Living (IADL) scale [33] consists of eight different items: ability to use the telephone, shopping, food preparation, housekeeping, laundry, mode of transportation, responsibility for own medications, and handling of finances. Each item was scored from 1 (no impairment) to 3 to 5 (severe impairment), which yielded a total range of 8 to 31 points. A mathematical correction of the sum of the IADL scores was performed to avoid gender-dependent activities affecting the result [34]. The Physical Self-Maintenance Scale (PSMS) [33] consists of six different items: toilet, feeding, dressing, grooming, physical ambulation, and bathing. Each item was scored from 1 (no impairment) to 5 (severe impairment), which allowed a total range of 6 to 30 points. Trained dementia nurses obtained the ADL evaluation from an interview with the caregiver. To facilitate the comparison of rates in MMSE, ADAS-cog, IADL, and PSMS scores, changes in score were converted to positive values, which were indicative of improvement, and negative values, which were indicative of decline.

### Statistical analyses

The IBM SPSS statistics software (version 18.0; SPSS Inc., Chicago, IL, USA) was used to perform the statistical analyses. The level of significance was defined as *P *< 0.05 if not otherwise specified. Observed-case analyses were performed to avoid overestimation of the treatment effect by imputing higher, previous outcome scores in a long-term study of a progressively deteriorating disease.

One-way analysis of variance (ANOVA) with Bonferroni correction was used to compare the differences among the means obtained for the three independent groups, a *t *test was performed to analyze two independent groups, and a χ^2 ^test was computed to analyze categorical variables.

Estimates of effect sizes were computed using Cohen's *d *(*d *= difference in group means/error SD_within_). Cohen's *d *was calculated as the difference between predicted means from the final mixed-effects model for a given pair of groups divided by the estimated within-group error standard deviation in the model.

#### Mixed models

Mixed, linear and nonlinear, fixed and random coefficient regression models [35] using "subject" as a hierarchical variable (that is, to allow correlation within subjects) were analyzed. The mixed models method also takes into account variations in the number of follow-up assessments available for the participants and unequal time intervals between the collected data points, which are common statistical limitations observed in longitudinal studies. The non-completers contributed information during the time of participation; thus, we considered the trajectories of all patients. Collinearity analyses of the variables included in the models showed no sign of multicollinearity, that is, the undesirable situation where one independent variable is a linear function of other independent variables. Model assumptions were checked using residual analyses.

Time was defined as the exact number of months between the baseline and each visit, thus using all data points at the correct time intervals. To adjust for baseline differences, the initial cognitive scores for each patient and their interaction with linear and quadratic terms for months in the study (to enable a nonlinear rate of decline in the models) were included as fixed effects, that is, Time in months (or Time in months^2^) × MMSE (or ADAS-cog) baseline score. Thus, the dependent variables were the cognitive scores assigned at the second and subsequent assessments for each patient; that is, the models do not intend to predict the scores at the start of ChEI treatment. The random terms in the models were an intercept and time in months, with an unstructured covariance matrix. Several sociodemographic and clinical background variables were also included as fixed effects. The predictors investigated were classical risk factors, such as age at first assessment (in years), the clinician's estimate of age at onset (in years), gender, years of education, carrier of the APOE ε4 allele, solitary living, functional ability, and number of medications at baseline. In addition, concomitant medications (antihypertensive/cardiac therapy, anti-diabetics, lipid-lowering agents, estrogens, nonsteroidal anti-inflammatory drugs (NSAIDs)/acetylsalicylic acid, antidepressants, antipsychotics, and anxiolytics/sedatives/hypnotics) were included. The impact of ChEI treatment was analyzed using the different drug agents and dosages. Finally, some biologically plausible interactions with cognitive severity at the start of treatment or with time in the study were included in the models, that is, gender, education, age, and functional ability at baseline. The terms "gender with carrier of APOE ε4 allele" and "type of ChEI with dose" were also included.

The ChEI agents were coded as a set of dummy variables. The dose could vary during the treatment period for an individual patient and between patients. Therefore, the mean dose used during the entire follow-up period was calculated for each patient. Furthermore, to obtain a similar metric of percent maximum dosage for the three ChEIs, the mean dose was divided by the maximum recommended dose for each drug, that is, 10 mg for donepezil, 12 mg for rivastigmine, and 24 mg for galantamine. The change of dosage between the assessments was also calculated using the percentage of maximum dose. Nonsignificant variables (*P *> 0.05) were removed in a backward stepwise elimination manner. The hierarchical principle was observed in these analyses; terms that appeared in interactions were not considered for elimination.

## Results

### Baseline characteristics

The demographic and clinical characteristics of the 843 patients, who were divided into groups corresponding to the three ChEI-agents, donepezil (*n *= 456, 54%), rivastigmine (*n *= 183, 22%), and galantamine (*n *= 204, 24%), are displayed in Table 1. The rivastigmine cohort exhibited a significantly smaller proportion of individuals living alone (22%) compared with the donepezil (38%) and galantamine (35%) groups (*P *< 0.001).

**Table 1:** Demographic and clinical characteristics

	Donepezil	Rivastigmine	Galantamine	Total subjects	*P-*value
Variable	*N *= 456/54%	*N *= 183/22%	*N *= 204/24%	*N *= 843	
Female gender	295/65%	106/58%	133/65%	534/63%	0.229
APOE ε4 carrier, (*n *= 829)	303/68%	119/66%	143/72%	565/68%	0.456
Solitary living at baseline	173/38%^a^	40/22%^b^	72/35%^a^	285/34%	< 0.001
Completion rate after three years	190/42%	85/46%	93/46%	368/44%	0.447
Antihypertensives/Cardiac therapy	177/39%	83/45%	70/35%	330/39%	0.096
Anti-diabetics	16/4%^a^	8/4%^a, b^	16/8%^b^	40/5%	0.048
Lipid-lowering agents	29/6%^a^	30/16%^b^	33/16%^b^	92/11%	< 0.001
Estrogens	38/8%	13/7%	8/4%	59/7%	0.124
NSAIDs/Acetylsalicylic acid	127/28%	65/36%	61/30%	253/30%	0.160
Antidepressants	114/25%	42/23%	53/26%	209/25%	0.754
Antipsychotics	26/6%^a^	4/2%^a, b^	3/2%^b^	33/4%	0.015
Anxiolytics/Sedatives/Hypnotics	63/14%	26/14%	24/12%	113/13%	0.750

Variable	Mean ± standard deviation (SD)				*P-*value

Estimated age at onset, years	72.6 ± 6.8^a^	71.6 ± 7.9^a, b^	70.9 ± 8.4^b^	71.9 ± 7.4	0.023
Estimated AD duration, years	3.1 ± 2.2	3.1 ± 2.5	2.9 ± 1.6	3.0 ± 2.1	0.380
Age at first assessment, years	75.7 ± 6.4^a^	74.6 ± 7.5^a, b^	73.7 ± 8.1^b^	75.0 ± 7.1	0.004
Education, years	9.3 ± 2.4^a^	9.0 ± 2.3^a^	10.0 ± 2.8^b^	9.4 ± 2.5	< 0.001
MMSE score at baseline	21.2 ± 3.8	21.6 ± 3.8	21.8 ± 3.6	21.4 ± 3.8	0.070
ADAS-cog score (0 to 70) at baseline	21.8 ± 8.8^a^	19.6 ± 8.9^b^	18.7 ± 8.7^b^	20.6 ± 8.9	< 0.001
IADL score at baseline	16.7 ± 5.5^a^	15.3 ± 5.1^b^	14.4 ± 5.3^b^	15.9 ± 5.4	< 0.001
PSMS score at baseline	7.6 ± 2.3^a^	7.4 ± 1.8^a, b^	7.1 ± 2.0^b^	7.4 ± 2.1	0.013
Number of medications at baseline	2.8 ± 2.3	3.0 ± 2.6	2.8 ± 2.5	2.8 ± 2.4	0.448
Mean dose of ChEI during the entire follow-up period, mg/day	7.1 ± 1.8	6.5 ± 2.1	16.1 ± 3.4		
Follow up-visits per subject	5.9 ± 1.8	6.1 ± 1.7	6.1 ± 1.7	6.0 ± 1.8	0.380

^a, b ^Results from *post hoc *tests (Bonferroni correction) are indicated by superscript letters (two groups with the same letter do not differ significantly within that variable).

Abbreviations: ADAS-cog, Alzheimer's Disease Assessment Scale-cognitive subscale; APOE, Apolipoprotein E; ChEI, cholinesterase inhibitors; IADL, Instrumental Activities of Daily Living scale; MMSE, Mini-Mental State Examination; NSAID, Nonsteroidal anti-inflammatory drugs; PSMS, Physical Self-Maintenance Scale.

Lipid-lowering agents were only used by 6% of the donepezil-treated subjects, whereas 16% of the patients in the other two cohorts were treated with this type of medication (*P *< 0.001). The usage of anti-diabetics and antipsychotics differed between the donepezil and the galantamine cohort: 4% vs 8% (*P *= 0.048) and 6% vs 2% (*P *= 0.015), respectively.

The donepezil-treated subjects had a higher mean age of onset of AD (F(2, 836) = 3.80, *P *= 0.023), were older (F(2, 840) = 5.69, *P *= 0.004), and exhibited a more impaired basic ADL ability (F(2, 825) = 4.40, *P *= 0.013) at the start of the ChEI treatment compared with the galantamine cohort. A higher level of education was found among the individuals treated with galantamine (F(2, 838) = 8.00, *P *< 0.001), whereas lower cognitive ability, as assessed using ADAS-cog scores (F(2, 824) = 10.32, *P *< 0.001) (but not using the MMSE), and more impaired instrumental ADL ability at baseline (F(2, 825) = 14.18, *P *< 0.001) were detected for the donepezil cohort compared with the other patients.

The three ChEI groups did not differ in gender, carrier status of APOE ε4 allele, completion rate after three years, medication use (antihypertensive/cardiac therapy, estrogens, NSAIDs/acetylsalicylic acid, antidepressants, and anxiolytics/sedatives/hypnotics), estimated duration of AD, MMSE baseline score, number of medications at baseline, or number of visits per subject.

No difference in MMSE or ADAS-cog scores at the start of ChEI treatment was detected regarding gender, presence of the APOE ε4 allele (no/yes), or usage of NSAID/acetylsalicylic acid therapy (no/yes). Male patients had significantly more years of education compared with females (mean ± SD, 9.7 ± 2.8 vs 9.2 ± 2.3 years; t(839) = 3.09; *P *= 0.003). A higher level of education was also observed for individuals carrying the APOE ε4 allele compared with non-carriers (9.6 ± 2.6 vs 9.1 ± 2.2 years; t(825) = -2.68; *P *= 0.005). No significant difference regarding mean years of education was found between those who used NSAID/acetylsalicylic acid therapy and those who did not. Carriers of the APOE ε4 allele were significantly younger at the start of ChEI treatment compared with non-carriers (74.2 ± 7.2 vs 76.4 ± 6.7 years; t(827) = 4.08; *P *< 0.001). Patients receiving NSAID/acetylsalicylic acid therapy were older than those not using this medication (77.4 ± 5.5 vs 73.9 ± 7.5 years; t(839) = -6.76; *P *< 0.001). No significant age difference was detected between genders.

To describe and compare the cognitive ability at baseline among patients with various ages and years of education, patients were divided into three subgroups according to age (≤ 70, 71 to 80, and > 80 years) and education (≤ 9, 10 to 12, and > 12 years). The oldest age group (> 80 years) was significantly more impaired than the other groups regarding its ADAS cog score of 22.4 ± 9.0 compared with 19.9 ± 9.5 for the ≤ 70 years group and 20.2 ± 8.5 for the 71 to 80 years group (F(2, 824) = 4.47, *P *= 0.012). Using the MMSE scale, there were no differences in baseline scores among the age groups. The group with the lowest level of education (≤ 9 years) had a significantly lower cognitive ability at baseline (MMSE, 21.1 ± 3.8; ADAS-cog, 21.2 ± 8.8) compared with the highest educated group (> 12 years) (MMSE, 22.9 ± 3.3; (F(2, 838) = 11.43; *P *< 0.001; and ADAS-cog, 17.6 ± 8.5; (F(2, 822) = 7.87; *P *< 0.001).

### Long-term outcomes

The MMSE mean difference from the baseline score (95% confidence interval (CI)) was -0.6 (-0.8 to -0.3) after one year of ChEI treatment, -2.3 (-2.7 to -1.9) after two years, and -3.2 (-3.7 to -2.7) after three years. The ADAS-cog mean difference from the baseline score (95% CI) was -1.8 (-2.3 to -1.3), -4.8 (-5.6 to -4.0), and -7.3 (-8.5 to -6.1), at one, two, and three years after the start of treatment, respectively. No differences were detected among the three ChEI agents.

#### ChEI dose

During the study, an increasing number of patients received higher doses of ChEI. After one year, the mean ± SD doses of donepezil, rivastigmine, and galantamine were 7.7 ± 2.5, 7.7 ± 2.9, and 18.8 ± 4.5 mg, respectively. After two years, they were 8.3 ± 2.4, 8.2 ± 2.9, and 19.4 ± 4.7 mg, respectively. Finally, after three years, the doses were 8.4 ± 2.4, 8.3 ± 2.7, and 20.0 ± 4.7 mg, respectively.

#### Dropout analyses

Overall, 56% of the patients who had at least three assessments did not complete the three-year study. The reasons for dropout from the study were: admission to nursing home (13%, *n *= 110), initiation of concomitant memantine therapy (8%, *n *= 66), poor effect/deterioration (6%, *n *= 48), death (5%, *n *= 44), withdrawal of informed consent (5%, *n *= 39), compliance problems (4%, *n *= 37), side effects (4%, *n *= 35), switching to another study (3%, *n *= 24), switching to another ChEI agent (2%, *n *= 18), somatic disease unrelated to ChEI treatment (2%, *n *= 17), and other reasons (4%, *n *= 35).

Table 2 shows that the completers exhibited significantly better cognitive and functional abilities at the start of the ChEI treatment compared with the non-completers (*P *< 0.001) and received a higher mean dose of ChEI during the study (*P *< 0.001). The other variables of interest in this study, such as gender, presence of the APOE ε4 allele, age at baseline, years of education, and usage of NSAIDs/acetylsalicylic acid, did not differ between the completers and those who discontinued the study.

**Table 2:** A comparison of the completer and non-completer groups

	Completers	Non-completers	*P-*value
Variable	*N *= 368/44%	*N *= 475/56%	
Female gender	64%	62%	0.614
APOE ε4 carrier	67%	69%	0.652
Estimated age at onset, years^a^	71.8 ± 7.4	72.1 ± 7.5	0.513
Age at first assessment, years^a^	74.9 ± 7.1	75.0 ± 7.2	0.744
Education, years^a^	9.4 ± 2.5	9.4 ± 2.5	0.978
MMSE score at baseline^a^	22.3 ± 3.4	20.7 ± 3.9	< 0.001
ADAS-cog score (0 to 70) at baseline^a^	18.2 ± 8.3	22.4 ± 8.9	< 0.001
IADL score at baseline^a^	14.5 ± 5.3	16.9 ± 5.2	< 0.001
PSMS score at baseline^a^	7.0 ± 1.7	7.8 ± 2.3	< 0.001
Number of medications at baseline^a^	2.8 ± 2.5	2.8 ± 2.3	0.827
NSAIDs/Acetylsalicylic acid	29%	31%	0.649
ChEI-dose^b^	70%	63%	< 0.001

^a ^Mean ± standard deviation

^b^Mean percentage of the maximum recommended dose, that is, 10 mg donepezil, 12 mg rivastigmine and 24 mg galantamine.

Abbreviations: ADAS-cog, Alzheimer's Disease Assessment Scale-cognitive subscale; APOE, Apolipoprotein E; IADL, Instrumental Activities of Daily Living scale; MMSE, Mini-Mental State Examination; NSAID, Nonsteroidal anti-inflammatory drugs; PSMS, Physical Self-Maintenance Scale.

In the multivariate mixed models, a better six-month response to ChEI therapy was observed for the completers using both MMSE and ADAS-cog scores as outcome variables (*P *= 0.001). However, the subsequent long-term rate of cognitive decline was not different between the completers and the non-completers. Adjustment for "dropout" (no/yes) as an additional independent variable in the models did not alter the outcome of the other significant predictor variables.

### Factors that affected the outcome

**Figure 1:**
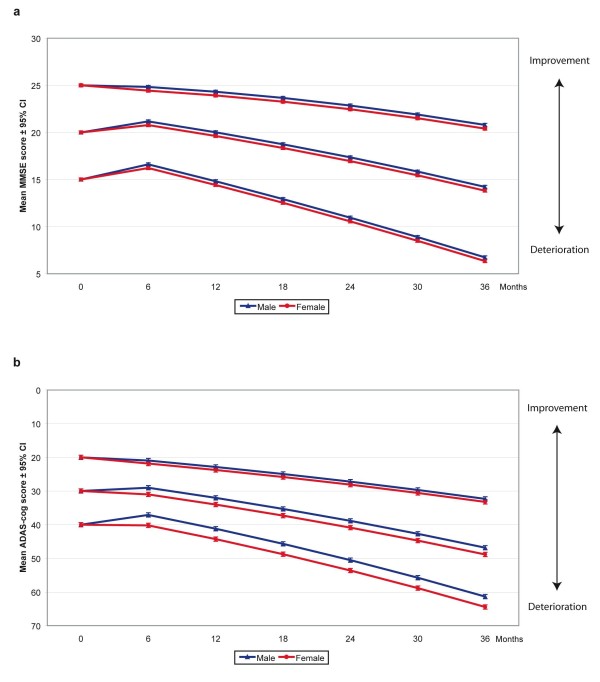
**Cognitive outcome and gender**. **a) **MMSE, prediction of outcome for different baseline scores divided by gender Three-year mean outcomes with 95% confidence intervals predicted by the mixed models for patients with different Mini-Mental State Examination (MMSE) scores (15, 20, and 25 were used as arbitrary examples), at the start of ChEI treatment and according to gender. Males demonstrated a better six-month treatment response compared with females (*P *= 0.010). The calculated outcomes were based on a 75-year-old patient who did not receive NSAID/acetylsalicylic acid treatment, had nine years of education, exhibited an IADL baseline score of 16, and received 65% of the maximum recommended dose of ChEI. **b**) ADAS-cog, prediction of outcome for different baseline scores divided by gender. Three-year mean outcomes with 95% confidence intervals predicted by the models for patients with different Alzheimer's Disease Assessment Scale-cognitive subscale (ADAS-cog) scores (20, 30, and 40 were used as arbitrary examples), at the start of treatment and according to gender. Male subjects showed a better response to treatment compared with females. An interaction effect of ADAS-cog baseline score × Gender was detected (*P *= 0.015), that is, the difference between genders increased with lower baseline scores. The calculated outcomes were based on a 75-year-old patient who was an APOE ε4 carrier, did not receive NSAID/acetylsalicylic acid treatment, had nine years of education, exhibited an IADL baseline score of 16, and received 65% of the maximum recommended dose of ChEI.

Mixed-effects (fixed and random, linear and nonlinear) models were performed (4,136 observation points) to identify the sociodemographic and clinical factors that affected the long-term MMSE and ADAS-cog outcomes. The models, significant predictors, and unstandardized β coefficients with 95% CI are presented in Table 3; the predicted mean scores with 95% CI are presented in Table 4. Estimates of effect sizes using Cohen's *d *for significant predictors in the final mixed models are presented in Table 5. Slower deterioration in cognitive ability was observed for patients with less cognitive impairment at baseline. Non-carriers of the APOE ε4 allele (ADAS-cog only) and patients receiving NSAID/acetylsalicylic acid therapy or a higher dose of ChEI (regardless of drug agent) exhibited a greater response to ChEI therapy after six months, with Cohen's *d *values ranging from 0.22 to 0.50, indicating small to medium effect sizes. The interaction effects of cognitive severity and age at baseline, time in months from the start of treatment, gender, and years of education showed that these variables cannot be interpreted separately. Male patients exhibited a greater response to ChEI treatment after six months compared with females, as measured using the MMSE scale, although the effect size was small (0.19) (Figure 1a). In addition, an interaction effect between gender and ADAS-cog score at baseline demonstrated that this difference and the magnitude of effects were more pronounced in subjects who were more cognitively impaired (Figure 1b). As an example, male individuals with a baseline ADAS-cog score of 40 responded, on average, 3.1 points better than females, and males with a baseline ADAS-cog score of 20 responded an additional 0.9 points better compared with females.

**Table 3:** Factors affecting the long-term outcome with MMSE or ADAS-cog score as dependent variables

	MMSE			ADAS-cog		
Percentage of variance accounted for, all fixed terms	53.7%, *P *< 0.001			57.8%, *P *< 0.001		
**Significant predictors in final mixed models**	**β**	**95% CI (β)**	***P-*value**	**β**	**95% CI (β)**	***P-*value**

**Fixed terms**						
Intercept	-25.766	-36.047, -15.484	< 0.001	-8.756	-19.030, 1.518	0.095
Time in months from baseline	-0.507	-0.605, -0.409	< 0.001	-0.211	-0.381, -0.040	0.016
MMSE (ADAS-cog) baseline score	2.666	2.074, 3.259	< 0.001	1.604	1.157, 2.051	< 0.001
MMSE (ADAS-cog) baseline score^2^	-0.018	-0.028, -0.008	< 0.001			ns
Time in months × MMSE (ADAS-cog) baseline score	0.023	0.019, 0.027	< 0.001	0.016	0.011, 0.021	< 0.001
Time in months^2 ^× MMSE (ADAS-cog) baseline score	-0.0001	-0.0001, -0.0001	< 0.001	0.0001	0.00004, 0.0002	0.004
***Background variables:***						
Gender (male = 0, female = 1)	-0.395	-0.718, -0.072	0.017	-1.290	-3.262, 0.681	0.199
MMSE (ADAS-cog) baseline score × Gender			ns	0.110	0.020, 0.199	0.016
APOE ε4 carrier (no = 0, yes = 1)			ns	1.072	0.239, 1.906	0.012
NSAIDs/Acetylsalicylic acid (no = 0, yes = 1)	0.440	0.094, 0.785	0.013	-1.037	-1.890, -0.184	0.017
Education, years	0.085	0.017, 0.153	0.014	-0.147	-0.339, 0.044	0.131
Time in months × Education, years	-0.013	-0.019, -0.007	< 0.001	0.018	0.003, 0.033	0.016
Age at first assessment, years	0.361	0.237, 0.485	< 0.001	0.168	0.036, 0.300	0.013
MMSE (ADAS-cog) baseline score × Age	-0.017	-0.023, -0.011	< 0.001	-0.012	-0.018, -0.006	< 0.001
IADL score at baseline	-0.090	-0.124, -0.056	< 0.001	0.256	0.170, 0.343	< 0.001
ChEI-dose^a^	0.010	0.001, 0.018	0.024	-0.040	-0.062, -0.019	< 0.001
**Random terms (variance)**						
Intercept	2.613	2.166, 3.153	< 0.001	13.887	10.274, 18.770	< 0.001
Time in months	0.027	0.023, 0.032	< 0.001	0.131	0.108, 0.158	< 0.001

Solitary living, concomitant medications with the exception of NSAIDs/Acetylsalicylic acid, age at onset, basic ADL ability, change of dosage and the variable comparing the ChEI agents were not significant.

β values were unstandardized and are expressed per one unit increase for continuous variables and for the condition present in dichotomous variables.

^a^Mean percentage of the maximum recommended dose, that is, 10 mg donepezil, 12 mg rivastigmine and 24 mg galantamine.

Abbreviations: ADAS-cog, Alzheimer's Disease Assessment Scale-cognitive subscale; APOE, Apolipoprotein E; ChEI, Cholinesterase inhibitors; CI, Confidence interval; IADL, Instrumental Activities of Daily Living scale; MMSE, Mini-Mental State Examination; NSAID, Nonsteroidal anti-inflammatory drugs; ns, not significant.

**Table 4:** Predicted mean scores from the mixed models (95% confidence interval)

	MMSE	ADAS-cog
Months in study		
6	21.6 (21.3, 21.8)	22.1 (21.4, 22.7)
12	20.6 (20.3, 20.8)	24.0 (23.3, 24.8)
18	19.4 (19.2, 19.7)	26.2 (25.4, 27.0)
24	18.2 (17.9, 18.5)	28.6 (27.8, 29.5)
30	16.8 (16.4, 17.2)	31.2 (30.2, 32.2)
36	15.3 (14.9, 15.7)	34.0 (32.9, 35.0)

Abbreviations: ADAS-cog, Alzheimer's Disease Assessment Scale-cognitive subscale; MMSE, Mini-Mental State Examination.

**Table 5:** Cohen's *d *effect size estimates for significant predictors in final mixed models

	MMSE				ADAS-cog^a^			
Time in months from start of ChEI treatment		6	12	36		6	12	36
**Pairs of groups**								
Males vs females^b^		0.19	0.19	0.19	ADAS-cog score 40	0.77	0.77	0.77
					30	0.50	0.50	0.50
					20	0.23	0.23	0.23
Age, 85 vs 65 years^b^	MMSE score 15	1.07	1.07	1.07	ADAS-cog score 40	1.55	1.55	1.55
	20	0.24	0.24	0.24	30	0.95	0.95	0.95
	25	-0.58	-0.58	-0.58	20	0.36	0.36	0.36
Age, 85 vs 75 years^b^	MMSE score 15	0.53	0.53	0.53	ADAS-cog score 40	0.78	0.78	0.78
	20	0.12	0.12	0.12	30	0.48	0.48	0.48
	25	-0.29	-0.29	-0.29	20	0.18	0.18	0.18
Age, 75 vs 65 years^b^	MMSE score 15	0.53	0.53	0.53	ADAS-cog score 40	0.78	0.78	0.78
	20	0.12	0.12	0.12	30	0.48	0.48	0.48
	25	-0.29	-0.29	-0.29	20	0.18	0.18	0.18
Education, 9 vs 15 years		-0.03	0.20	1.10		-0.06	0.10	0.75
Education, 12 vs 15 years		-0.01	0.10	0.55		-0.03	0.05	0.38
Education, 9 vs 12 years		-0.01	0.10	0.55		-0.03	0.05	0.38
APOE ε4, non-carrier vs carrier		ns	ns	ns		0.27	0.27	0.27
NSAIDs/Acetylsalicylic acid therapy, yes vs no		0.22	0.22	0.22		0.25	0.25	0.25
ChEI-dose, 100% vs 50%^c^		0.24	0.24	0.24		0.50	0.50	0.50

^a ^To facilitate comparisons of effect sizes, the plus/minus sign is reversed for ADAS-cog.

^b^Due to the interaction effects ADAS-cog baseline score × Gender, MMSE baseline score × Age and ADAS-cog baseline score × Age, effect sizes are presented for MMSE scores of 15, 20 and 25 and for ADAS-cog scores of 20, 30 and 40, which are used as arbitrary examples.

^c^Mean percentage of the maximum recommended dose, that is, 10 mg donepezil, 12 mg rivastigmine and 24 mg galantamine.

Abbreviations: ADAS-cog, Alzheimer's Disease Assessment Scale-cognitive subscale; APOE, Apolipoprotein E; ChEI, Cholinesterase inhibitors; MMSE, Mini-Mental State Examination; NSAID, Nonsteroidal anti-inflammatory drugs; ns, not significant.

**Figure 2:**
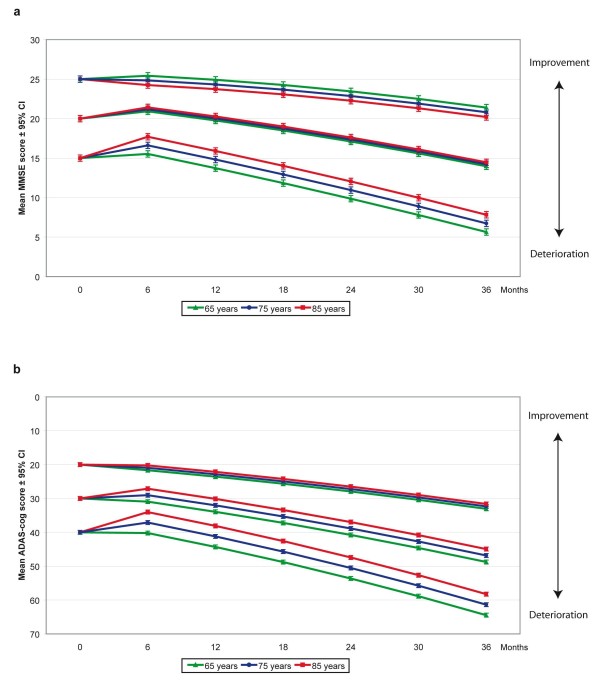
**Cognitive outcome and age**. **a) **MMSE, prediction of outcome for different baseline scores and ages. Three-year mean outcomes with 95% confidence intervals predicted by the mixed models for patients with different Mini-Mental State Examination (MMSE) baseline scores (15, 20, and 25) and ages (65, 75, and 85 years), used as arbitrary examples. Older subjects with a baseline MMSE score < 22 exhibited a better six-month treatment response compared with younger patients (*P *< 0.001). In addition, the interaction MMSE score × Age at the start of ChEI treatment showed a more pronounced age difference at lower baseline scores (*P *< 0.001). The calculated outcomes were based on a male patient who did not receive NSAID/acetylsalicylic acid treatment, had nine years of education, exhibited an IADL baseline score of 16, and received 65% of the maximum recommended dose of ChEI. **b**) ADAS-cog, prediction of outcome for different baseline scores and ages. Three-year mean outcomes with 95% confidence intervals predicted by the models for patients with different Alzheimer's Disease Assessment Scale-cognitive subscale (ADAS-cog) baseline scores (20, 30, and 40) and ages (65, 75, and 85 years), used as arbitrary examples. Older individuals exhibited a better response to treatment compared with younger subjects (*P *= 0.043). The interaction ADAS-cog score × Age at the start of treatment showed a greater age difference at lower baseline levels (*P *< 0.001). The calculated outcomes were based on a male patient who was an APOE ε4 carrier, did not receive NSAID/acetylsalicylic acid treatment, had nine years of education, exhibited an IADL baseline score of 16, and received 65% of the maximum recommended dose of ChEI.

Older individuals exhibited a better response to treatment compared with younger subjects, if they had MMSE scores < 22 at baseline (Figure 2a) and through all levels of ADAS-cog score (Figure 2b). The interaction Cognitive ability × Age at the start of treatment exhibited a greater age difference and larger effect sizes (0.53 to 1.55) for patients with more cognitive severity. For example, 85-year-old individuals with a baseline MMSE score of 15 responded on average 2.2 points better than 65-year-old individuals, and 85-year-old individuals with a baseline ADAS-cog score of 40 responded an additional 6.2 points better compared with 65-year-old individuals after six months of ChEI treatment. Moreover, there was an interaction effect between years of education and time in the study. Differential dropout over time did not cause this effect, as no difference regarding mean years of education was detected for patients with different numbers of assessments (F(5, 835) = 1.56; *P *= 0.168). A higher level of education implied increased cognitive impairment over time, with a magnitude of effects of 0.38 to 1.10 after three years. As an example, a subject with 15 years of education exhibited on average an additional 2.2 points of MMSE and 3.0 points of ADAS-cog deterioration after three years compared with an individual with nine years of education.

If not otherwise specified, the arbitrary examples of patients presented in the figures were based on an average male that was aged 75 years, was a carrier of the APOE ε4 allele, did not receive NSAID/acetylsalicylic acid therapy, had nine years of education, exhibited an IADL score of 16, and received 65% of the maximum recommended dose of ChEI.

The background variables solitary living, concomitant medications (with the exception of NSAIDs), age at onset, basic ADL ability, type of ChEI agent, change of dosage and the interaction effects, Gender × Carrier of APOE ε4 allele, and Type of ChEI × Dose were not significant when included in the mixed models. The percentages of variance accounted for in the dependent variable, regarding all fixed predictors, were 53.7% for MMSE and 57.8% for ADAS-cog, which implies a good fit of the models (*P *< 0.001).

## Discussion

Using mixed models, we found that a higher mean dose of ChEI, male gender, older age, NSAID/acetylsalicylic acid therapy, and absence of the APOE ε4 allele were predictors of a better short-term ChEI-treatment response and long-term outcome. The type of ChEI did not influence the results. The patients that were more severely impaired cognitively exhibited a better response to ChEI therapy, but declined faster subsequently. Individuals with a lower level of education showed a slower cognitive decline. These findings were similar for both the MMSE and ADAS-cog scales; however, ADAS-cog is more sensitive in detecting effects, which gives credibility to the results. For example, the graded effects of baseline cognitive ability with gender or with age were observed more clearly using the ADAS-cog scale and had larger effect sizes.

Our SATS cohort reflects the alteration of patient characteristics and treatment of AD over more than one decade. During the years that ChEI treatment has been available, the patient population has evolved to become younger, better educated, and exhibit less disease severity at baseline. The prescription of lipid-lowering agents has become more common, whereas antipsychotics have been less used, as more patients seek care and treatment at an earlier stage of AD. In this study, these differences were observed between the donepezil cohort enrolled earlier and the galantamine subjects included later. Similar changes were described in other long-term studies [36] and show the need for using advanced multivariate methods, such as mixed models, to compensate adequately for differences and effects of interactions or time between the treatment cohorts.

The rate of disease progression varies among AD patients; however, the knowledge on prognostic factors is limited [37]. In the present study, a faster deterioration in cognition was observed for the patients that were more severely impaired after their initial response to treatment. A more rapid decline in ADL performance in individuals with lower cognitive ability was also described in a recent study from our group [34]. Moreover, in this study, a better cognitive response to treatment was observed among males, which was in agreement with the multivariate results obtained in a three-month study of tacrine and galantamine [23]. A lower percentage of males was also described among the rapid progressors in a longitudinal study of progression rate [38]. Inconsistently, a review of sex influences on ChEI treatment in AD found that a clear relation was not established between gender and response to therapy. The possible sex differences reported in that review were small and exhibited large individual variation; thus, this subject requires further investigation. The morphological brain differences between genders or sex hormones are theories that could explain this dissimilar response to treatment [39].

Older age was a predictor of a better treatment response in the current study, whereas the subsequent rate of cognitive deterioration was not related to age. However, an interaction effect between age and cognitive severity was identified. The oldest patients (> 80 years) in this study were more cognitively impaired at baseline and exhibited a marked positive response to ChEI therapy; however, severity, and not age, predicted a faster long-term progression. In contrast, the younger-age group (< 65 years) showed greater improvement in a three-month donepezil study that used a univariate analysis [25]. However, the patients had a somewhat lower mean cognitive ability compared with that of our cohort, and the analysis did not adjust for that factor, which could influence the outcome (as discussed above). A recent meta-analysis model of AD progression reported the absence of a significant impact of age; however, the distribution of the mean age in the model was narrow [40]. Other studies found a faster rate of cognitive decline in younger individuals [15,37]. It is reasonable to assume that AD progresses more rapidly when the disease is detected at younger ages, as hereditary and more aggressive variants of the disease may have a greater influence on the outcome [37].

In the present study, the individuals with the highest education (> 12 years) were less cognitively impaired at their baseline assessment, which is consistent with the patient characteristics described in a recent paper on progression rate [38]. A higher level of education was associated with faster cognitive deterioration in this study, as well as in several other reports [15,16,41], and with faster ADL decline, as reported in a previous study from our group [34]. Bennett *et al*. [42] suggested that the association between senile plaques and the level of cognitive function varies according to years of education, as it appeared that more education provides some form of cognitive reserve. Furthermore, in accordance with this "brain-reserve hypothesis" [41], subjects with more years of education are expected to have higher cognitive ability during adulthood, thus requiring a relatively greater burden of pathology when dementia is clinically evident [42]. Nevertheless, some studies found inconsistent results or no association between the level of education and the rate of cognitive decline. Years of education or age had no significant effects in a multivariate comparison of ChEI- and memantine-treated patients, performed by Atri *et al*. [29]; however, the measures of dispersion in that cohort were small compared with those of our study. In contrast to the results of the current study, the group of slow pre-progressors observed by Doody *et al*. [38] had a higher level of education, but this variable was not a significant predictor of longer-term ADAS-cog outcome. The high value of mean years of education (approximately 13 to 14 years) reported in these American cohorts [29,38] suggests a more narrow selection of patients compared with the sample included in the SATS (mean, 9.4 years of education). In Sweden, the health system is publicly funded and the income or insurance coverage of individuals is rarely an issue when seeking care [43].

In line with the results of this study regarding APOE genotype, Martins *et al*. [44] used a mixed model with nonlinear terms and observed that the presence of at least one APOE ε4 allele may precipitate the rate of cognitive decline. Conflicting evidence regarding whether the ε4 allele influences disease progression was found in other studies that used linear models [17,20]. Nonlinear models proved to fit the data better compared with linear models in Martins' study [44]; moreover, the mixed models method also takes the individual variability into account, which increases the variance explained to a larger extent. Unlike some studies of response to tacrine, which exhibited inconsistent associations between APOE genotype and gender, an open-label trial of donepezil demonstrated an absence of significant differences between the responses of ε4-carriers and non-carriers [24].

Interestingly, divergent results concerning the relationship between AD progression and NSAID treatment have been discussed and this potential connection remains unresolved. In epidemiological studies, NSAIDs exhibited neuroprotective effects, suggesting a greater reduction in risk of AD with longer use of these drugs [45]. The Rotterdam study showed that a reduction in risk was only observed after the first two years of cumulative NSAID therapy [46] and the US Veterans study reported a marked decrease in the odds ratio for AD after four years of NSAID usage [47]. In contrast to our naturalistic study, the two randomized trials reported most recently, an 18-month [48] and a 12-month [49] study, found no beneficial effect of NSAID treatment vs placebo on cognitive response in AD populations. It is possible that these trials did not include a follow-up time that was sufficient for a protective effect to emerge compared with the longer perspective of the SATS. Longitudinal naturalistic studies with more detailed information regarding the specific NSAIDs used, dosing, and so on, are needed to investigate further this potentially important finding. Knowledge of the factors that cause differences in outcome is essential for a better understanding of AD and its rate of progression.

Our study, as well as most previous publications comparing the three ChEI agents, showed no difference in effect on cognitive outcome among the drugs [11,12]. However, higher doses of ChEIs were associated with a more positive long-term cognitive outcome in the present study, which is in agreement with the results of a meta-analysis of randomized trials, as the latter showed that larger ChEI doses were related to a larger effect [50]. Theoretically, if we assumed that the patients received 100% of the maximum recommended ChEI dose, instead of the average 65% observed in the SATS, our model would estimate a six-month mean response to therapy of 4.0 ADAS-cog points, instead of 2.6 points. Treatment with a higher dose of ChEI was also related to significant delays in nursing-home placement [51,52]. These results suggest the importance of using adequate ChEI doses in AD therapy.

The advantages of the SATS are the well-structured and prospective assessments of a large number of ChEI-treated AD patients in routine clinical settings. Recognized scales are administered in a uniform manner across all centers. The scheduled six-month visits and access to a responsible contact nurse for each subject represent security, continuity, and good quality of care. The three-year completion rate of 44% obtained for the present cohort is high compared with other AD extension or naturalistic studies. Most prior publications report 20% to 39% completers after three years [53-55]. The high dropout rate in long-term AD studies may contribute to greater mean cognitive scores for the patients remaining in the study, assuming that they benefit more from ChEI therapy. Our results showed that the completers received a higher mean dose of ChEI during the study, suggesting a better tolerance of the treatment. In the models, the outcomes of the non-completers were also included during their time of participation. Other than the lower cognitive and functional abilities at baseline observed for the non-completers, which the multivariate mixed models took into account, those patients were similar to the completers regarding the other characteristics. The reasons for dropout in long-term AD studies are complex and may vary considerably. For example, dropout caused by nursing-home placement might depend not only on the worsening of AD, but also on somatic diseases or changes in the health status of the caregiver.

The SATS is an open-label, nonrandomized study that might have variations between the treatment cohorts, which were not addressed by the model variables. The fact that placebo-controlled designs are not permitted (because of ethical concerns) is a limitation of AD therapy studies longer than six months; therefore, no control group was enrolled in the SATS. The presence of behavioral, psychotic, and extrapyramidal symptoms was not recorded in this study; these are factors that have been reported as affecting the rate of decline [28]. To compensate somewhat for this limitation, the use of psychiatric medications was included in the models; however, these variables exhibited no significant effect on outcome.

The ability to predict and distinguish overall outcomes would provide clinicians and the social services with better tools to estimate the disease prognosis, manage the patients, and plan for the future. It is important to recognize and treat patients with a better probability of response or a more aggressive course of AD as early as possible [56]. Knowledge and awareness of critical characteristics that may influence the response to, and outcome of, pharmaceutical trials are important. To improve the management of patients and enhance the efficacy of ChEI therapy and its cost benefits, it is essential to understand factors that influence response to treatment and longitudinal outcome in a routine clinical setting. For example, the patients that had more cognitive impairment in our study exhibited a better response to therapy, stressing the importance of not excluding this group from treatment opportunities.

## Conclusions

In conclusion, this study showed that male gender, older age, absence of the APOE ε4 allele, and NSAID/acetylsalicylic acid treatment or a higher mean dose of ChEI were predictors of better response to ChEI therapy and of a more favorable longitudinal outcome. Lower cognitive ability at baseline was a predictor of improved response to ChEI treatment. The long-term outcome was better for patients with a higher cognitive level at the start of therapy or for less-educated individuals. The demographic and clinical composition of the AD cohort under study may be one of the explanations for the heterogeneity of results observed in different studies. Future studies are warranted to investigate differences in response to treatment and longitudinal outcome based on various patient characteristics. Long-term protective effects, such as the possible impact of NSAIDs or other protective treatments, may take years to develop. The knowledge gained from naturalistic ChEI treatment studies will continue to be important.

## Abbreviations

AD: Alzheimer's disease; ADAS-cog: Alzheimer's Disease Assessment Scale-cognitive subscale; ADL: activities of daily living; APOE: apolipoprotein E; ChEI: cholinesterase inhibitors; CI: confidence interval; DSM-IV: *Diagnostic and Statistical Manual of Mental Disorders*: 4^th ^edition; IADL: Instrumental Activities of Daily Living Scale; MMSE: Mini-Mental State Examination; NSAIDs: NonSteroidal Anti-Inflammatory Drugs; PSMS: The Physical Self-Maintenance Scale; SATS: Swedish Alzheimer Treatment Study; SD: standard deviation

## Competing interests

The authors declare that they have no competing interests.

## Authors' contributions

CW participated in the study, supervised the data collection, was responsible for the statistical design and for carrying out the statistical analyses, interpreted the results, and drafted the paper. AKW and EL participated in the study, assisted in the analysis and interpretation of the data, and critically revised the manuscript. LM designed the study and critically revised the manuscript. All authors read and approved the final manuscript.
